# Investigating the Synergistic Interaction of Diabetes, Tobacco Smoking, Alcohol Consumption, and Hypercholesterolemia on the Risk of Pancreatic Cancer: A Case-Control Study in Italy

**DOI:** 10.1155/2014/481019

**Published:** 2014-04-29

**Authors:** Giuseppe La Torre, Antonella Sferrazza, Maria Rosaria Gualano, Chiara de Waure, Gennaro Clemente, Agostino Maria De Rose, Nicola Nicolotti, Gennaro Nuzzo, Roberta Siliquini, Antonio Boccia, Walter Ricciardi

**Affiliations:** ^1^Sapienza university of Rome, Piazzale Aldo Moro 5, 00185 Rome, Italy; ^2^Institute of Public Health, Catholic University of the Sacred Heart, Rome, Italy; ^3^Department of Public Health Sciences, University of Turin, Turin, Italy; ^4^Department of Surgery, Hepatobiliary Unit, Catholic University of the Sacred Heart, Italy

## Abstract

The aims of the present research are to investigate the possible predictors of pancreatic cancer, in particular smoking status, alcohol consumption, hypercholesterolemia, and diabetes mellitus, in patients with histologically confirmed pancreatic carcinoma and to examine the synergism between risk factors. A case-control study (80 patients and 392 controls) was conducted at the Teaching Hospital “Agostino Gemelli” in Rome. A conditional logistic regression was used for the statistical analysis and results were presented as odds ratio (OR) and 95% confidence intervals (95% CI). We also investigated the possible interactions between risk factors and calculated the synergism index (SI). The multivariate analysis revealed that hypercholesterolemia and alcohol consumption resulted in important risk factors for pancreatic cancer even after the adjustment for all variables (OR: 5.05, 95% CI: 2.94–8.66; OR: 2.25, 95% CI: 1.30–3.89, resp.). Interestingly, important synergistic interactions between risk factors were found, especially between ever smoking status and alcohol consumptions (SI = 17.61) as well as alcohol consumption and diabetes (SI = 17.77). In conclusion, the study confirms that hypercholesterolemia and alcohol consumption represent significant and independent risk factors for pancreatic cancer. Moreover, there is evidence of synergistic interaction between diabetes and lifestyle factors (drinking alcohol and eating fatty foods).

## 1. Background

Pancreatic cancer is a relative uncommon disease and will remain a challenging problem in the 21st century [1]. The last international cancer statistics report that it is at the 13th position for annual incidence and the 10th for mortality rate worldwide [2]. In particular, in the developed countries, it represents the ninth most common cancer in both sexes and the fifth and fourth for mortality rates, in males and females, respectively [3]. In addition, it is one of the most aggressive cancers, with an overall 5-year survival rate of less than 5% [4].

To date, aetiology of pancreatic cancer is largely unknown and no screening tests for early detection are available. The identification of risk factors could provide a tool for primary prevention leading to reduce the burden of disease. Among risk factors of this cancer, there is evidence that tobacco smoking, alcohol abuse, hypercholesterolemia, and diabetes play an important role. However, there is no evidence on the synergistic effect of these risk factors combined.

Tobacco smoking is a major risk factor of pancreatic cancer [4]. Epidemiological studies underline a strong association between pancreatic tumor and smoking status [5].

Carcinogens derived from tobacco smoke probably reach the pancreas via the bloodstream after being absorbed from the lungs or from the upper aerodigestive tract. In addition, there is a possibility that ingested tobacco products reach the pancreas directly after reflux into the pancreatic ductal system from the duodenum [6]. In particular, nicotine, the major component in cigarette smoke, could be implicated in pancreatic danger, causing alteration in the signal transduction pathways and in the expression of protooncogene in pancreatic cells [7].

A recent meta-analysis [8] has estimated a risk of pancreatic cancer (RR) of 1.74 (95% CI: 1.61–1.87) for current smokers and of 1.20 (95% CI: 1.11–1.29) for former ones.

Alcohol abuse is one of the most common causes of acute pancreatitis that is the predominant cause of chronic pancreatitis. The latter is a major risk factor for the development of pancreatic cancer [9]. To consider alcohol consumption as a risk factor for pancreatic cancer is still controversial: some authors found a positive association [10–14] while others found an inverse correlation [15, 16]. Moreover it is important to consider that the smoking status could be a confounder for this association [17]. Recent data from pooled analyses in consortia involving multiple case-control and cohort studies suggest that heavy (but not moderate or light) alcohol consumption also may increase pancreatic cancer risk [18].

Another important risk factor for developing pancreatic cancer is metabolic syndrome: a recent meta-analysis has concluded that metabolic syndrome and its components, such as hypercholesterolemia, are associated with higher risk of this type of malignancy [19].

In several papers, the association with diabetes mellitus has been studied but further studies are needed to clarify if diabetes mellitus represents a risk factor or a consequence of pancreatic tumour [20]. In a case-control study, people with diabetes for more than 5 years before a diagnosis of pancreatic tumour have RR = 2.07 (95% CI: 1.02–4.20). The risk of pancreatic cancer was 6.21 (95% CI: 1.61–23.96) for patients treated with insulin and 1.21 (95% CI: 0.50–2.92) for those treated with oral hypoglycemic drugs [21]. Given this context, the aims of the present research were

- to investigate the possible predictors of pancreatic cancer, in particular smoking status, alcohol consumption, hypercholesterolemia, and diabetes mellitus, in patients with histologically confirmed pancreatic carcinoma;
- to examine the synergism between risk factors.

## 2. Methods

### 2.1. Patients and Setting

A case-control study was conducted at the Teaching Hospital “Agostino Gemelli” in Rome. The inclusion criteria for cases were being a patient with histologically confirmed pancreatic carcinoma, resected in the Department of Surgery-Hepatobiliary Unit, during the period 2005–2008. Sample size calculations were based on the following assumptions:

- prevalence of cigarette smoking among general population in 2003: 27.6% (ISTAT 2006);
- association between smoking and pancreatic cancer: RR = 2.10 (Does quality of observational studies affect the results of a meta-analysis?): the case of cigarette smoking and pancreatic cancer [22];
- rate between cases and controls = 1 : 5, in order to get sufficient power.

Using these parameters, we calculated with Epi Info the need to recruit 80 cases and 400 controls. According to the methodology used in previous studies [23, 24], the controls, with no diagnosis of cancer, were recruited at the same hospital selecting randomly outpatients without a diagnosis of cancer. Those controls were matched for age (±5 years) and gender with cases, in a ratio 5 to 1. The study participants were selected and directly interviewed by well-trained interviewers. All the participants gave their informed consent to participate and, according to the Italian law, a notification of the study was transmitted to the local ethical committee.

### 2.2. Data Collection

Information on the following variables was collected through patient history questions during the first clinical examination, in particular

- type-II diabetes diagnosed before the interview;
- smoking habits (current smoking, former smoking, or never smoking);
- alcohol consumption (drink of at least a glass of wine, beer, or hard liquor per day).
- hypercholesterolemia;
- age and gender.

The presence in the patient history of diabetes and hypercholesteremia was subsequently confirmed by laboratory parameters and/or medication.

### 2.3. Statistical Analysis

Descriptive statistics was performed using frequencies, percentages, frequency tables for qualitative variables, and mean ± standard deviation (SD) for quantitative variables.

For the univariate analysis Mann-Whitney and chi-square tests were used. Differences in frequencies between the cases and controls were evaluated by chi-square test and Fisher exact probability test.

In order to investigate the risk factors for pancreatic cancer, a conditional logistic regression model was used, following a multiplicative model, and results were presented as odds ratio (OR) and 95% confidence intervals (95% CI). Variables entering the model were selected according to the Hosmer and Lemeshow procedures [25].

Concerning the smoking habits, cases and controls were classified as ever smokers (current or former smokers) and no smokers, while the alcohol consumption was classified as no drink versus at least one drink/day.

The synergism index was calculated as follows: *S* = [OR11 − 1]/([OR01 + OR10] − 2), where OR11 is equal to OR of the joint effect of two risk factors and OR10 and OR01 are equal to OR of each risk factor in the absence of the other. A value of *S* equal to unity was interpreted as indicative of additivity, whereas a value greater than unity was indicative of superadditivity and synergism [26, 27].

Finally, the population attributable risk percentage (PAR%) for each statistically significant risk factor was calculated using the adjusted OR of that factor and its prevalence in the control group (Pe) [28].

Statistical significance level was set at *P* = 0.05. Statistical analyses were performed using the statistical software Stata release 9.0.

## 3. Results

Eighty cases and 392 controls were analysed. Among matched patients 56.3% were males. The mean ages of cases and controls were, respectively, 63.6 and 64.8 years. As shown in Table 1, statistically significant differences for having a pancreatic cancer emerged for diabetes mellitus: in fact it appeared that diabetic patients had a higher probability to be affected by pancreatic cancer (*P* = 0.025).

For the variables ever smoking, alcohol consumption, and hypercholesterolemia, there were statistically significant differences (*P* = 0.007, *P* < 0.001, and *P* < 0.001, resp.): ever smokers, drinkers, and patients with hypercholesterolemia had a higher likelihood to develop a pancreatic cancer.

As shown in the Table 2, hypercholesterolemia and alcohol consumption resulted in important risk factors for pancreatic cancer even after the adjustment for all variables (OR: 5.05, 95% CI: 2.94–8.66; OR: 2.25, 95% CI: 1.30–3.89, resp.).

Using the prevalence of pancreatic risk factors in the controls and the estimated adjusted ORs of them, we estimated that the PAR% values explained by the diabetes mellitus, ever cigarette smoking, alcohol consumption, and hypercholesterolemia in our study population were 10.9, 8.6%, 13.9%, and 29.8%, respectively.

In Table 3 the joint effect of ever cigarette smoking, alcohol consumption, diabetes mellitus, and hypercholesterolemia on pancreatic cancer risk was shown. It is clear that there was an indication for the additivity and synergism between risk factors, especially between ever smoking status and alcohol consumptions, as well as alcohol consumption and diabetes.

## 4. Discussion

Our study investigated risk factors of a relative uncommon cancer and went deep in the synergic association between well known risk factors of pancreatic cancer. Results of our multivariate analysis confirmed that alcohol consumption and hypercholesterolemia are significant risk factors for pancreatic cancer.

Regarding the study of the interactions between variables, it clearly showed that there was an indication for the additivity and synergism between risk factors, especially between ever smoking status and alcohol consumptions, as well as alcohol consumption and diabetes.

Tobacco smoking is universally reported as an environmental risk factor for pancreatic cancer and accounts for approximately 25% of all pancreatic cancers [29]. Smoking always should be measured and adjusted for in etiologic epidemiologic studies of pancreatic cancer and encouraging nonsmoking should reduce the incidence of the disease [29, 30].

Furthermore, the significant association between diabetes and pancreatic malignancy was reported in several studies [21, 31–35] and in a meta-analysis also [36]. In our study, risk for pancreatic cancer was demonstrated almost twofold higher among patients affected by diabetes and around threefold among smokers, but the crude odds ratios are not confirmed at the multivariate analysis (Table 2), suggesting the presence of factors confounding the analysis.

In our study alcohol consumption appears to be associated with pancreatic cancer, in line with results of previous studies [37–39].

Interestingly, the higher risk of pancreatic cancer seems to be related to hypercholesterolemia. This finding is in accordance with a case-control study and a meta-analysis investigating the association of metabolic syndrome and pancreatic cancer [19].

Additionally, we estimated that if the diabetes mellitus, cigarette smoking, alcohol consumption, and hypercholesterolemia are associated with pancreatic cancer independently of each other, each factor contributed to 10.9, 8.6%, 13.9%, and 29.8% of the pancreatic cancer cases in this study, respectively. In a study conducted by Hassan et al. [28] similar results were found concerning diabetes mellitus and ever smoking. About alcohol consumption, results are not comparable because the authors considered only the heavy alcohol consumption. Talamini et al. in 2010 found that tobacco smoking and alcohol drinking are two independent risk factors for pancreatic cancer which may be responsible for around 33% of these cancers [40].

Moreover, a clear indication for the additivity between these risk factors, especially between ever smoking status and alcohol consumptions as well as alcohol consumption and diabetes, was shown in our research.

Few studies have analyzed the potential interaction between smoking and other risk factors, focusing particularly on genetic ones [41, 42] and concluding that further investigations are needed in this direction. To date, no studies investigated the interaction between diabetes mellitus, hypercholesterolemia, and alcohol consumption.

In interpreting our results the main limitations of our study should be acknowledged. Firstly, given the study design, a risk of underreporting and recall bias has to be addressed: these factors can give an underestimation of the prevalence of drinkers and smokers. Secondly, we have to consider that information about family history of pancreatic cancer is lacking. A further limitation consists in the lacking of information about use of aspirin [43, 44], physical activity [45], and reproductive factors [46, 47], as well as date of onset and type of medication for diabetes mellitus. Moreover, using the synergistic effect, there is no practical solution to sample sizing considering at least two risk factors simultaneously. However, we really believe that the case-control study was sufficiently powered looking at the results of Table 2, and no selection bias should be due to this aspect.

Finally, the use of a hospital-based case-control study design may overestimate the prevalence of exposure for some risk factors, such as smoking, diabetes mellitus, and alcohol consumption. However, if this was the case, even if misclassification of exposure occurred, our results could give OR towards the null, and the synergistic hypothesis would be confirmed.

On the contrary, a strength of this study was the statistical analysis: we performed a conditional logistic regression model in order to analyze matched data. Moreover, this study adds knowledge on synergism between risk factors for pancreatic cancer that is a little explored topic.

In conclusion, the present study confirms that, after controlling for possible confounding factors, hypercholesterolemia and alcohol consumption represent significant and independent risk factors for pancreatic cancer. Moreover, there is evidence of synergistic interaction between diabetes and lifestyle factors (drinking alcohol and eating fatty foods).

We believe that identifying individuals at the highest risk for pancreatic cancer could be a great opportunity in order to develop prevention programmes and early detection of pancreatic cancer.

## Figures and Tables

**Table 1:** Characteristics of cases and controls.

Variables (*N* = 320)	Cases (%)	Controls (%)	*P* ^∧^
Gender			
Males	45 (56.3)	204 (52)	0.492
Females	35 (43.8)	188 (48)	
Age, mean (SD)	63.59 (11.05)	64.92 (10.95)	0.319*
Diabetes mellitus			
Yes	19 (23.8)	54 (13.8)	**0.025**
No	61 (76.3)	338 (86.2)	
Ever smoking			
Yes	34 (42.5)	107 (27.3)	**0.007**
No	46 (57.5)	285 (72.7)	
Alcohol consumption			
Yes	41 (51.3)	103 (26.3)	**<0.001**
No	39 (48.7)	289 (73.7)	
Hypercholesterolemia			
Yes	40 (50)	56 (14.3)	**<0.001**
No	40 (50)	336 (85.7)	

^∧^Chi-square test.

*Mann-Whitney test.

**Table 2:** Adjusted odds ratios of the selected variables.

Variables (*N* = 320)	Crude OR (95% CI)	Adjusted OR (95% CI)*
Gender		
Males (reference)	1	1
Females	1.04 (0.78–1.40)	1.16 (0.81–1.65)
Age	0.99 (0.97–1.01)	0.99 (0.96–1.01)
Diabetes		
No	1	1
Yes	**1.95 (1.08–3.52)**	1.64 (0.84–3.21)
Hypercholesterolemia		
No	1	1
Yes	**6.00 (3.56–10.11)**	**5.05 (2.94–8.66)**
Ever smoking		
No	1	1
Yes	**1.97 ** **(1-20–3.23)**	1.28 (0.72–2.29)
Alcohol consumption		
No	1	1
Yes	**2.95 (1.80–4.83) **	**2.25 (1.30–3.89)**

*Adjusted for all variables.

**Table 3:** Synergistic interaction between ever smoking and diabetes, alcohol consumption and diabetes, and alcohol consumption and ever smoking.

Variables (*N* = 320)		AOR (95% CI)*	Synergistic interaction
Ever smoking	Diabetes		
No	No (reference)	1	**4.93 **
Yes	No	1.42 (0.84–2.41)	
No	Yes	1.19 (0.57–2.48)	
Yes	Yes	4.01 (1.63–9.88)	
Ever smoking	Hypercolesterol		
No	No (reference)	1	**1.32 **
Yes	No	0.86 (0.47–1.58)	
No	Yes	4.00 (2.16–7.41)	
Yes	Yes	4.78 (2.47–9.24)	
Alcohol consumption	Ever smoking		
No	No (reference)	1	**17.61**
Yes	No	1.48 (0.78–2.78)	
No	Yes	0.65 (0.30–1.43)	
Yes	Yes	3.29 (1.89–5.73)	
Alcohol consumption	Diabetes		
No	No (reference)	1	**17.77**
Yes	No	1.48 (0.78–2.78)	
No	Yes	0.65 (0.30–1.43)	
Yes	Yes	3.31 (1.50–7.32)	
Alcohol consumption	Hypercolesterolemia		
No	No (reference)	1	**1.30**
Yes	No	1.41 (0.81–2.50)	
No	Yes	3.75 (2.01–6.98)	
Yes	Yes	5.11 (2.66–9.82)	
Diabetes	Hypercolesterolemia		
No	No (reference)	1	**3.68**
Yes	No	0.61 (0.25–1.48)	
No	Yes	3.65 (2.10–6.35)	
Yes	Yes	9.31 (3.72–23.33)	

*AOR: odds ratio adjusted for work activity, diabetes mellitus, ever smoking, and alcohol consumption.
